# Hummingbird-Leaves-Reared Black Soldier Fly Prepupae: Assessment of Nutritional and Heavy Metal Compositions

**DOI:** 10.3390/biology9090274

**Published:** 2020-09-05

**Authors:** Giva Kuppusamy, Chee Kei Kong, Ganeswaran Chandra Segaran, Eliyarajan Tarmalingam, Max Herriman, Mohd Fathil Ismail, Tahir Mehmood Khan, Liang Ee Low, Bey-Hing Goh

**Affiliations:** 1 Crops for the Future Research Centre, The University of Nottingham Malaysia Campus, Jalan Semenyih, Semenyih 43500, Selangor, Malaysia; maxherriman@gmail.com (M.H.); fathil1786@gmail.com (M.F.I.); 2 Innovation and Technology Advancement Department, GK Aqua Sdn Bhd, Lot 563 Kg Sg Machang Ulu, Lenggeng 71750, Negeri Sembilan, Malaysia; ganeswaran@myhindupage.org (G.C.S.); gk.aqua35@gmail.com (E.T.); 3 Biofunctional Molecule Exploratory (BMEX) Research Group, School of Pharmacy, Monash University Malaysia, Bandar Sunway 47500, Selangor, Malaysia; cheekei92@gmail.com (C.K.K.); tahir.khan@uvas.edu.pk (T.M.K.); zack_1129@hotmail.com (L.E.L.); 4 Institute of Pharmaceutical Sciences (IPS), University of Veterinary & Animal Sciences (UVAS) Out Fall Road, Lahore 54000, Pakistan; 5 Institute of Pharmaceutics, College of Pharmaceutical Sciences, Zhejiang University, 866 Yuhangtang Road, Hangzhou 310058, China; 6 College of Pharmaceutical Sciences, Zhejiang University, 866 Yuhangtang Road, Hangzhou 310058, China

**Keywords:** black soldier fly, *Sesbania grandiflora*, nutrition, insect meal, agriculture

## Abstract

Black soldier fly (BSF) larva is an attractive animal feed replacer due to its noticeable nutritional content. However, the conventional rearing method often resulted in BSF with undesirably high heavy metal residues that are harmful to animals. In this work, putrefied *Sesbania grandiflora* (*S. Grandiflora*) leaves were employed as feed to rear BSF larvae. The resultant BSF prepupae were found to contain 43.5% protein and 16.7% fat, reflecting a comparable protein content and a 2-fold reduction in crude fat than those reared using conventional kitchen waste. Moreover, high quantities of arginine (25.4 g/kg dry matter basis (DM)), carnitine (32.9 g/kg DM), and short-chain fatty acids, including lauric (40.00%), palmitic (19.20%), and oleic (12.10%) acids, have also been noticed in the BSF prepupae. Furthermore, the BSF larvae have been recorded with 0.185 mg/kg chromium, 0.380 mg/kg selenium, and mercury below the detection limit, which is far lower than those reared using conventional kitchen and agricultural wastes (≈1.7 mg/kg chromium, 1.2 mg/kg selenium, and 0.2 mg/kg mercury). Overall, the study shows that the nutritional quality of BSF prepupae is extensively improved when using *S. Grandiflora* as their feed. The resultant BSF prepupae may serve as an alternative feed for animal rearing.

## 1. Introduction

Insects are known to contain high fat and crude protein content (>30% on dry matter basis), making them desirable as a replacement source for animal feed production [1]. Black soldier fly (BSF), or *Hermetia illucens*, is a nonpest insect distributed around the tropics and warm temperate regions [2]. The prepupae of BSF have been recorded to contain 42% protein, 35% fat, and common essential amino and fatty acids [3]. Furthermore, BSF larvae can consume a wider range of decomposing organic material at a quicker and more efficient rate than other insects due to the presence of numerous enzymes, namely, leucine arylamidase, α-galactosidase, β-galactosidase, α-mannosidase, and α-fucosidase, in their body, which enable them to digest glycoproteins, glycolipids, and carbohydrates [4]. Thus, BSF is considered a promising ingredient for animal feed production since it can effectively convert organic wastes (kitchen and agricultural wastes) into edible biomass, such as protein and fat [5,6,7]. However, these conventionally utilized wastes for BSF rearing often resulted in BSF larvae with a high heavy metal content [8,9]. Concerns have been raised over heavy metal accumulation in the larvae and prepupae, which may pass through the food chain as animal feed and eventually cause toxicity to humans.

Better larva quality is essentially dependent on the type, quantity, and nutritional quality of the substrate/food provided [10]. However, considering the diet cost of the larva, the desirable choice of substrate would be a sustainable and underutilized crop that may ultimately produce high-quality larval biomass. *Sesbania grandiflora*, commonly known as sesbania, vegetable hummingbird, and agate, is an interesting underutilized crop that could be used as a rearing substrate for BSF larvae. It is a small, erect, fast-growing, and sparsely branched tree that belongs to the *Fabaceae* family. *S. grandiflora* is native to tropical Asia and is commonly available in Malaysia [11]. The leaves of *S. grandiflora* are known for a high content of essential amino acids, minerals, vitamins, and other active compounds [12]. It has previously been employed as a partial meal replacer for fish in the tilapia fish diet, and it has demonstrated good survival, growth, and feed utilization [13]. More importantly, *S. grandiflora* was found to have the capability to eliminate heavy metals [14].

In this study, the putrefied *S. grandiflora* leaves were exploited as growing substrates for BSF larvae. The nutritional composition, amino acid profile, and fatty acids profile of both fresh and putrefied *S. grandiflora* leaves, as well as the prepupae fed with *S. grandiflora* putrefied leaves, were examined. The heavy metal contamination was also assessed in the prepupae fed with *S. grandiflora* putrefied leaves. This work demonstrates a promising and cost-effective biowaste treatment approach that can effectively convert underutilized crops into secondary raw materials (e.g., animal feed) with improved nutritional value.

## 2. Materials and Methods

### 2.1. Sesbania Grandiflora Leaf Putrefaction

**Figure 1:**
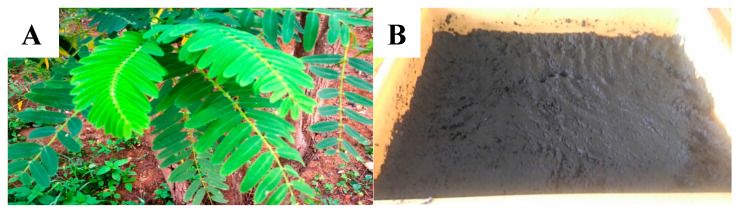
(**A**) Fresh *S. grandiflora* leaves; (**B**) Putrefied *S. grandiflora.*

Fresh *S. grandiflora* cuttings were obtained and identified in the Crops for The Future Field Research Centre (CFF), Malaysia (Figure 1A). The stem was first manually separated from the leaves and chopped using a blender to obtain a 1-mm edible size for the larvae. Then, it was mixed with water at the ratio of 5:1 to guarantee the optimal moisture content level (30–40%, to promote aerobic reaction) for larvae growth and preparation of the leaves. It was allowed to naturally putrefy for a period of two weeks at room temperature in trays (Figure 1B). Three samples of fresh and putrefied substrate were collected and dried in an oven for 5 h at 80 °C until a constant weight was obtained before being vacuum-packed and sent to SGS (Malaysia) Sdn. Bhd. for chemical analysis. Both fresh and putrefied *S. grandiflora* leaves were analyzed for their nutritional contents, but only putrefied *S. grandiflora* leaves were fed to the larvae due to the higher nutritional content obtained from the analysis.

### 2.2. Rearing and Harvesting

BSF flies were collected from the wild and then bred in an indoor insectarium facility of CFF, Malaysia, via the methods described previously [15]. BSFs were attracted from the wild by the given substrate, and the flies were allowed to mate naturally. Their eggs are normally laid in between the given sticks and were carefully scraped from the wooden sticks using a scalpel. The eggs of BSF are about 1 mm long and creamy yellow in color. The collected eggs (0.20 g) were then carefully transferred and placed in triplicates on three 50 × 30 cm trays in the shade. Under optimum conditions of around 25–27 °C, they take approximately four days to incubate and hatch. The substrate was limited to a 7–8 cm depth to prevent excess heat and lack of oxygen, as well as the settlement of the substrate at the bottom of the tray due to the wiggling movement of the larvae. The tray was covered with a fine-mesh lid with a ventilation hole to maintain room temperature. The larvae were then subjected to a feeding regime of 50 g of putrefied leaves for the first three days, followed by 300 g daily until day 30 (the time period of the larvae to reach prepupal stage). Water was added to the substrate on a daily basis to maintain moisture (30–40%, to promote aerobic reaction) and to promote continuous aerobic degradation. The prepupae were collected from the tray at the end of the growth period as they tend to self-harvest by climbing to a drier place. Total prepupae biomass was recorded per tray after harvesting. The collected prepupae were washed with tap water and kept frozen at −20 °C overnight. Then, they were oven-dried for 24 h at 106 °C until a constant weight was obtained. Finally, the dried prepupae were grounded into fine particles and packed in vacuum, pending chemical analyses.

### 2.3. Nutritional Composition Analyses

Different analytical parameters such as moisture, crude protein, crude fat, crude fiber, and ash content were examined in accordance with AOAC official methods of analysis [16]. Moisture content was determined by using the conventional oven loss-of-drying method (991.02). The moisture content was expressed as a mass fraction in percentage. Protein content was estimated through the determination of nitrogen by the Kjeldahl method (984.13). Crude protein of substrates and prepupae were estimated by multiplying the total N with 6.25. According to Finke [17], this is a universally accepted factor for estimating the true protein in most of the insect species. Crude fat was determined gravimetrically through the solvent extraction method where a sample was extracted with diethyl ether, and the solvent was then removed by distillation using the Soxhlet system (945.16). The residue obtained from evaporation was dried and weighed to calculate the crude fat content. For crude fiber analysis, the sample was first defatted and treated successively with sulfuric acid, followed by sodium hydroxide of specified concentrations, known as the acid-base hydrolysis (962.09). Crude ash was determined through the decomposition of organic matter from a test portion by incineration at 550 °C for 3 h in a muffle furnace and weighing the final ash obtained (942.05). All experiments were performed in three replicates (*n* = 3).

### 2.4. Amino Acid Composition Analysis

The amino acid profile of fresh and putrefied substrates, as well as prepupae fed with the putrefied substrate, was carried out by high-performance liquid chromatography (HPLC), following the methods of AOAC (994.12). Prior to amino acid analysis, protein samples need to be acid-hydrolyzed to obtain stable amino acids. Oxidation was done to preserve cysteine and methionine, and base hydrolysis was done to preserve tryptophan. The experiment was performed in three replicates (*n* = 3).

### 2.5. Fatty Acid Composition Analysis

The fatty acid compositions of fresh and putrefied substrates, as well as prepupae fed with putrefied substrate, were determined by gas–liquid chromatography (GLC). For the determination of fatty acid composition, the samples were methylated and esterified by BF_3_-methanol esterification, following the methods of AOCS Ce 2-66 and 1-26 [18]. The experiment was performed in three replicates (*n* = 3).

### 2.6. Heavy Metal Analysis

Mercury levels of fresh and putrefied substrates and prepupae fed with putrefied substrate were determined by atomic absorption spectroscopy (AAS), where the sample is digested with concentrated sulfuric and nitric acids at 80 °C, followed by oxidation with potassium permanganate and potassium persulfate at 30 °C. Whereas chromium was analyzed using inductively coupled plasma optical emission spectrometry (ICP-OES), selenium was quantified through inductively coupled plasma (ICP-OEP).

### 2.7. Statistical Analysis

All data were analyzed statistically using the GNU PSPP sofware (https://www.gnu.org/software/pspp/) for statistical analysis of sampled data. One-way analysis of variance (ANOVA) was applied to analyze the nutritional composition of the harvested prepupae, which includes moisture, crude protein, crude fat, crude fiber, and ash content. A value of *p* < 0.05 was considered statistically significant.

## 3. Results and Discussion

### 3.1. Larval Development and Prepupae Yield

All harvested prepupae were of uniform size as the egg incubation time was synchronized, promoting a similar feed consumption rate. Generally, 7.5 mg clutches of egg incubated in a tray were recorded to provide approximately 3850 larvae, but due to a 10% mortality, only 3400 larvae survived, with an average weight of 0.1 g each. Thus, only 340 g of prepupae were harvested from each tray on day 30.

### 3.2. Nutritional Analysis

The nutritional value of fresh and putrefied *S. grandiflora* leaves is shown in Table 1. The nutritional analysis revealed that the putrefied leaves contained a higher amount in their moisture, crude protein, crude fat, crude fiber, and ash content compared to fresh leaves. There was an increase of 15 % in moisture, 17 % crude protein, 45 % crude fat, 11 % crude fiber, and 44 % ash in the putrefied *S. grandiflora* leaves compared to fresh leaves. Like fermentation, putrefaction increased the overall bioavailability of nutritional values in *S. grandiflora* leaves. The moisture content increase after putrefaction could be attributed to water absorption from the water added intermittently to the leaves to enable them to decay aerobically. Moisture is a prerequisite for putrefaction as water helps arrest bacteria and allows microbial, chemical, and enzymatic actions to take place in nature [19]. The crude protein concentration of fresh and putrefied *S. grandiflora* leaves was 29.94 ± 0.09% and 36.19 ± 0.12%, respectively. The protein content reported from this study was higher than those obtained in previous studies [20,21]. The microbially mediated process of putrefaction involves anaerobic bacteria consuming and digesting complex plant proteins. Undigested proteins or amino acids are consumed by putrefactive bacteria as a large protein molecule and then broken down into a simpler form that can be easily absorbed by the BSF larvae. The increased protein content after putrefaction was the putrefactive metabolites (ammonia and nitrogenous matter), measured as total Kjeldahl nitrogen.

Fiber is the indigestible part of carbohydrates found in all plants, and it is an important nutrient for ruminant livestock and humans. The fiber content in food may increase, decrease, or remain unchanged during exposure to different processing and storage methods [22]. A slight increase in the fiber content (0.87%) in putrefied *S. grandiflora* leaves was observed, which is due to the breakdown of dietary fiber polysaccharides by the putrefactive microorganisms. The mechanism behind this is most likely an enzymatic or acid breakdown to hydrolyze and metabolize insoluble polysaccharides. On the other hand, ash is useful for judging the nutritional characteristics of the feed because ash generally contains constant element composition. Ash content in plants, however, is not very good as a nutritional indicator because it varies widely, and the element composition depends on the soil and fertilizers. The overall improvements in the nutritional contents suggest that aerobically putrefied *S. grandiflora* leaves are a better alternative protein source than fresh *S. grandiflora* leaves, and, therefore, they were used to feed the BSF larvae.

BSFs could contribute a higher number of growth cycles per year and has a greater potential for protein yield compared to other insects as its life cycle is relatively short [23]. The quality of food fed to BSFs is important as it influences the larval development time and nutrient composition [24]. According to Oonincx et al. [25], larval development time increased when BSFs are fed with a low-protein diet (over five weeks) compared to a high-protein diet (three weeks). BSF larvae grown on substrates lower in proteins have a longer developmental time, are smaller in size, and have higher fat content [26]. Hence, a substrate with higher protein and lower fat content would be preferred for the industrial-scale production of BSFs.

In this study, BSF larvae fed with putrefied leaves contained 43.5% crude protein, reflecting an overall 1.2-fold increment in protein content as compared to the putrefied substrate. Such improvement in the protein level obtained is rather similar to the content reported by Diener et al. [27], ranging from 28.2% to 42.5% depending on the feeding regime. Another study also reported that the prepupae protein content was found to be 44%, and the crude fat was 33% [28]. A more recent study demonstrated lower protein content (33.45%) in BSF pupae fed with spent coffee grounds [29]. Contrarily, crude fat and ash content of the BSFs obtained from this study were lower compared with those reared on other organic wastes. The ethyl extract (crude fat) and ash content of BSFs reared on vegetable waste, chicken feed, and restaurant waste were 37.10% (317 g/kg DM) and 9.60% (96 g/kg DM), 33.60% (336 g/kg DM) and 10.00% (100 g/kg DM), and 38.60% (386 g/kg DM) and 2.70% (27 g/kg DM), respectively [30]. Comparative studies of the nutritive value of BSFs reared on chicken manure, brewers’ spent grain, and kitchen waste revealed that ash content, crude protein content, and crude fat were 9.3 ± 1.8 to 11.6 ± 0.5%, 33.0 ± 1.0 to 41.3 ± 0.5%, and 30.1 ± 0.4 to 34.3 ± 0.4%, respectively [31]. Insects have the ability to convert carbohydrates into lipids [30]. The crude fat content of BSFs may vary depending on the protein and carbohydrate content of the diet fed, with higher crude fat obtained if the feed is of high protein and carbohydrate content [32]. It should be noticed that the presence of too much fat in the substrate is also detrimental to the BSF larvae production cycle because the larvae would have difficulty breaking down the fat during the metamorphosis processes into an adult fly [10].

### 3.3. Amino Acid Analysis

BSFs can be considered an excellent source of energy and digestible amino acids [33]. The protein content of the substrate influences the availability of amino acids of the BSFs as it is the sole source of amino acids [34]. Based on Table 2, it is noteworthy that a contrasting amino acid profile of fresh and putrefied *S. grandiflora* leaves was observed. There was a drastic reduction in some of the amino acids in putrefied leaves, such as arginine, aspartic acid, carnitine, histidine, methionine, serine, tryptophan, tyrosine, and valine. However, the level of amino acids, including alanine, cysteine, glutamic acid, glycine, proline, isoleucine, leucine, lysine, phenylalanine, proline, and threonine, increased in the putrefied leaves. Asparagine and glutamine were not detected in all samples, including the larvae. Putrefied *S. grandiflora* leaves showed a higher level of the nonessential amino acid proline (5.10 ± 0.01 g/kg). Glycine was not detected in fresh *S. grandiflora* leaves. Similar to the protein metabolism by putrefactive bacteria, free amino acids can be further metabolized through bacterial proteolysis, a catabolic pathway involving highly specific enzymes that perform deamination and decarboxylation of amino acids, leading to anaerobic degradation. Certain amino acids (arginine, histidine, methionine) are preferentially reduced, while others are preferentially oxidized [35].

In the present study, the most abundant amino acids found in the larvae were arginine and carnitine, which were 25.41 ± 0.12 and 32.86 ± 0.02 g/kg DM, respectively. This value is close to that reported by Mwaniki and coworkers [36], which is 2.79 % (27.9 g/kg) for BSFs fed on a corn–soybean diet; the amino acid level in defatted BSF meal has up to 2.56 % (25.6 g/kg) [37]. Moreover, Meneguz et al. [38] also reported a lower level of arginine when fed with a chicken diet (20.3 g/kg DM), digestate (20.3 g/kg DM), vegetable waste (20.0 g/kg DM), and restaurant waste (19.9 g/kg DM) compared to larvae fed with putrefied *S. grandiflora* leaves, which was observed as around 25.4 g/kg DM in this study. Notably, levels of arginine and carnitine reported in prepupae fed with putrefied *S. grandiflora* leaves increased 5-fold and 3-fold, respectively, when compared to the levels of arginine and carnitine in putrefied *S. grandiflora* leaves alone. A much lower value was observed in BSFs reared on chicken manure (1.1 ± 1.8 g/kg), brewers’ spent grain (5.0 ± 4.3 g/kg), and kitchen waste (2.5 ± 2.2 g/kg), suggesting that the rearing substrate greatly affects the amino acid compositions of the prepupae [31]. Up to date, nonessential amino acid carnitine has not been found to be included in any of the studies related to BSFs. Interestingly, it is worth noticing that the methionine level in prepupae fed with putrefied *S. grandiflora* leaves was found to be 10-fold higher than the level found in putrefied *S. grandiflora* leaves.

Levels of proline, tryptophan, and valine were found to be very low at 0.71 ± 0.05, 0.93 ± 0.00, and 1.02 ± 0.05 g/kg DM in prepupae fed with putrefied *S. grandiflora*. The finding was in contrast to that obtained from BSFs reared on chicken manure, brewers’ spent grain, and kitchen waste, with values ranging from 1.2 ± 2.2 to 9.3 ± 0.8 g/kg [31]. The content of proline, tryptophan, and valine ranged from 5.4 to 28.2 g/kg in BSFs fed with chicken diet, digestate, vegetable waste, and restaurant waste [30].

### 3.4. Fatty Acid Analysis

Overall, the composition of most saturated and nonsaturated fatty acids was higher in putrefied leaves compared to fresh leaves (Table 3). Considerable differences between fresh and putrefied *S. grandiflora* leaves were observed, especially the reduction of linoleic acid from 21.30% to 9.27%, linolenic acid from 2.80% to 2.43% and arachidonic acid from 31.60% to 8.40% in putrefied leaves. Fresh leaves exhibited a high amount of palmitic (20.20 ± 0.19%), linoleic (21.30 ± 0.18%), and arachidonic (31.60 ± 0.31%) acids, whereas putrefied leaves exhibited the highest amount in palmitic acid (35.50 ± 0.20%), followed by oleic acid (16.90 ± 0.10%), and stearic acid (13.80 ± 0.07%).

In this study, the fatty acid composition of the rearing substrates did not directly affect the fatty acid of the prepupae. The fatty acid profile of the harvested prepupae was dominated by saturated fatty acids dominated by high C12:0 lauric acid (40.00 ± 0.90%) content, even only trace amounts of this fatty acid was found in its substrate. This suggests that C12:0 in BSFs might be synthesized from other nutrients present in the substrate, such as carbohydrates (starch and sugars) [30]. Other fatty acids present in noticeable amounts were C16:0 palmitic acid, C18:0 oleic acid, and C14:0 myristic acid, which made up 19.20 ± 0.20%, 12.10 ± 0.20%, and 11.10 ± 0.15%, respectively. The results were in agreement with earlier findings [30,38], but contradicted findings reported by Barroso et al. [39], Liland et al. [40], and Shumo et al. [31], and there were major differences in the fatty acid content of BSFs fed with different substrates at different levels.

The composition of crude fat directly influences the BSF fatty acid composition. BSF larvae fed with *S. grandiflora* were found to have very low omega-3-α-linolenic acid (ALA), beyond the detection limit. Thus, C18:3 rich fat could be included in the feed to create added value. According to St-Hilaire and coworkers [41], the fat content of BSF prepupae can be manipulated to contain desirable fatty acids such as ALA, EPA, and DHA. In this regard, a sustainable source of omega 3 fatty acids, such as marine algae and phytoplankton, can be added into the substrate to supplement the prepupae quality [42].

### 3.5. Heavy Metal Analysis

Concern over the bioaccumulation of pollutants in the larvae has been studied. For instance, heavy metals acquired from the rearing substrates may accumulate in the larvae or prepupae of BSFs [8]. The prepupae fed with putrefied *S. grandiflora* were detected to have different levels of bioaccumulation of some important heavy metals. Mercury was found to be lower than the detected limit, whereas chromium and selenium were found to be 0.185 and 0.380 mg/kg, respectively (Table 4). BSF larvae fed with solid aquaculture waste showed higher mercury, chromium, and selenium content, i.e., 0.2 ± 0.01, 1.6 ± 0.43 to 1.7 ± 0.56, and 1.01 ± 0.16 to 1.2 ± 0.26 mg/kg, respectively [43]. Heavy metals in aquatic systems can be attributed to the large volumes of wastewater, sewage, stormwater, and effluents from the thousands of industries that are shed into the wetlands, naturally produced by leaching from soil/rock to water, and introduced by human activities [44].

It is noteworthy that heavy-metal-contaminated soils are often nutrient-poor, which may limit the establishment of plants. On the other hand, plants possess mechanisms that involved in the detoxification of heavy metals in three ways: reduced uptake or increased efflux by pumping metals out of the plasma membrane through the chelation of metals by various ligands, such as phytochelatins, and the repair of stress damaged proteins [45]. Moreover, the *S. grandiflora* plant has been proven to be tolerant to heavy metals compared to other plants in in-vivo (whole plant) and in-vitro (tissue culture) studies by Ibrahim and Yousir [46].

Based on a study by Diener et al. [7], a high concentration of heavy metals, such as zinc and selenium, was found in substrates. Therefore, in this study, we have taken the heavy metal toxicity analysis as a serious concern. Based on the analysis in this study, the content of mercury, selenium, and chromium was found to be below toxicity levels and safe for use. According to the U.S. Food and Drug Administration (FDA), the maximum permissible limits of mercury, chromium, and selenium is 0.5, 0.2, and 0.3 ppm (mg/kg), respectively [47]. Hence, the BSF larvae grown on this safe and sustainable substrate, the underutilized crops of *S. grandiflora*, were found to be devoid of any mercury and chromium, with slightly excessive selenium. Selenium is an essential trace element in the diet of humans and domesticated animals and often added to the feed of animals in doses up to 0.3 ppm to prevent selenium deficiency diseases in farm animals or livestock [48]. Acute selenosis occurs when selenium content is higher than 20–30 ppm, whereas chronic and subacute selenium poisoning occurs at selenium doses below 3–5 ppm [49].

## 4. Conclusions

BSF larvae have the immense potential of being used as animal feed as they possess a high content of proteins, amino acids, and many important fatty acids. Underutilized crops are thought to be a safer and more cost-effective feeding choice for BSFs than kitchen and agricultural wastes. Our results demonstrate that the use of *S. grandiflora* as a substrate for BSFs can lead to the production of potential animal feeds (BSF prepupae), with 43.5% protein and 16.7% fat, which revealed a similar protein content and a 2-fold reduction in fat content compared to the BSFs reared on conventional kitchen and agriculture wastes. Moreover, these BSFs have also been recorded to possess high-quality amino acids and fatty acids while ensuring a substantial decrease in chromium (9-fold), selenium (3-fold), and mercury (undetected) content than those prepared conventionally. To achieve industrial-scale production of BSF larvae while maintaining a relatively constant nutrient profile, the production line would require a constant supply of substrates, possibly with a fairly constant chemical composition. Fortunately, *S. grandiflora* can be established and grows much more rapidly than other common tree legumes (e.g., *Calliandra, Gliricidia,* and *Leucaena*), which would make frequent replanting and industrial-scale BSF production possible [50]. It is believed that the utilization of the as-prepared BSF larvae in animal diets will benefit animal production. Future studies should be performed by investigating the effect of BSF larvae on the diet plans of different animal species for production.

## Figures and Tables

**Table 1:** Nutritional composition of *S. grandiflora* (fresh and putrefied) and prepupae fed with putrefied *S. grandiflora*.

Macronutrient (%)	Fresh *S. grandiflora*	Putrefied *S. grandiflora*	Prepupae Fed with Putrefied *S. grandiflora*
**Ash**	6.78 ± 0.09	12.03 ± 0.20	13.95 ± 0.25
**Crude fat**	4.35 ± 0.09	7.98 ± 0.08	16.70 ± 0.20
**Crude fiber**	7.36 ± 0.09	8.23 ± 0.12	9.57 ± 0.31
**Crude protein**	29.94 ± 0.09	36.19 ± 0.12	43.50 ± 0.23
**Moisture**	72.60 ± 0.12	85.07 ± 0.18	63.60 ± 0.00

Values presented in mean (g/kg or % dry matter) ± standard error (*n* = 3). No significant difference among the nutritional composition.

**Table 2:** Amino acid content of *S. grandiflora* (fresh and putrefied) and prepupae fed with putrefied *S. grandiflora*.

Amino Acid (g/kg)	Fresh *S. grandiflora*	Putrefied *S. grandiflora*	Prepupae Fed with Putrefied *S. grandiflora*
Alanine	0.49 ± 0.00	0.80 ± 0.00	6.58 ± 0.20
Arginine	11.0 ± 0.01	5.02 ± 0.00	25.41 ± 0.12
Asparagine	N.D.	N.D.	N.D.
Aspartid acid	2.14 ± 0.00	0.74 ± 0.00	8.04 ± 0.28
Carnitine	24.54 ± 0.03	10.30 ± 0.00	32.86 ± 0.02
Cystine	1.22 ± 0.00	1.85 ± 0.00	N.D.
Glutamic acid	2.99 ± 0.00	3.95 ± 0.00	9.63 ± 0.01
Glutamine	N.D.	N.D.	N.D.
Glycine	N.D.	0.26 ± 0.00	1.24 ± 0.07
Histidine	18.87 ± 0.02	14.57 ± 0.02	11.21 ± 0.15
Isoleucine	1.29 ± 0.00	1.36 ± 0.00	2.10 ± 0.14
Leucine	2.99 ± 0.00	3.55 ± 0.00	7.56 ± 0.05
Lysine	1.77 ± 0.01	1.88 ± 0.00	6.58 ± 0.19
Methionine	1.41 ± 0.00	0.45 ± 0.00	4.51 ± 0.00
Phenylalanine	N.D.	N.D.	2.15 ± 0.08
Proline	1.25 ± 0.00	5.10 ± 0.01	0.71 ± 0.05
Serine	3.42 ± 0.00	3.05 ± 0.00	10.83 ± 0.20
Threonine	4.60 ± 0.00	5.42 ± 0.00	9.11 ± 0.02
Tryptophan	0.32 ± 0.00	0.21 ± 0.00	0.93 ± 0.00
Tyrosine	5.42 ± 0.00	2.47 ± 0.00	6.33 ± 0.59
Valine	1.08 ± 0.00	0.52 ± 0.00	1.02 ± 0.05

Values presented in mean ± standard error (*n* = 3). N.D. = not detected (*p* < 0.1), where the mean is lower than the detection limit value.

**Table 3:** Fatty acid composition of *S. grandiflora* (fresh and putrefied) and prepupae fed with putrefied *S. grandiflora*.

Fatty Acid Composition (%)	Fresh *S. grandiflora*	Putrefied *S. grandiflora*	Prepupae Fed with Putrefied *S. grandiflora*
Arachidic C20:0	31.60 ± 0.31	8.40 ± 0.06	1.50 ± 0.00
Capric C10:0	0.50 ± 0.00	0.53 ± 0.17	1.30 ± 0.00
Caproic C6:0	0.40 ± 0.00	0.60 ± 0.00	0.10 ± 0.00
Caprylic C8:0	0.55 ± 0.30	0.80 ± 0.00	0.10 ± 0.00
Lauric C12:0	1.10 ± 0.00	1.60 ± 0.00	40.00 ± 0.90
Linoleic (alpha) C18:2	21.30 ± 0.18	9.27 ± 0.09	3.10 ± 0.05
Linolenic C18:3	2.80 ± 0.49	2.43 ± 0.19	N.D.
Myristic C14:0	1.50 ± 0.00	3.43 ± 0.03	11.10 ± 0.15
Oleic C18:1	8.50 ± 0.09	16.90 ± 0.10	12.10 ± 0.20
Palmitic C16:0	20.20 ± 0.19	35.50 ± 0.20	19.20 ± 0.45
Palmitoleic C16:1	1.10 ± 0.00	6.67 ± 0.12	8.50 ± 0.30
Stearic C18:0	10.40 ± 0.09	13.80 ± 0.07	3.20 ± 0.10

Values presented in mean ± standard error (*n* = 3). N.D. = not detected (*p* < 0.1), where the mean is lower than the detection limit value.

**Table 4:** Heavy metal content of prepupae fed with putrefied *S. grandiflora*.

Heavy Metal (mg/kg)	Prepupae Fed with Putrefied *S. grandiflora*
Chromium	0.185
Mercury	N.D.
Selenium	0.380

N.D. = not detected (*p* < 0.1), where the mean is lower than the detection limit value. LOD (limit of detection) <0.01 ppm or mg/kg.
